# Supplementary material for: Co-Treatments of Gardeniae Fructus and Silymarin Ameliorates Excessive Oxidative Stress-Driven Liver Fibrosis by Regulation of Hepatic Sirtuin1 Activities Using Thioacetamide-Induced Mice Model
Source: Antioxidants (Basel). 2022 Dec 30;12(1):97. doi: 10.3390/antiox12010097 (PMC9854785; doi:10.3390/antiox12010097)
Supplement: Supplementary file 1 [file antioxidants-12-00097-s001.zip › Supplementary Figures.pdf]

Supplementary Figure S1.

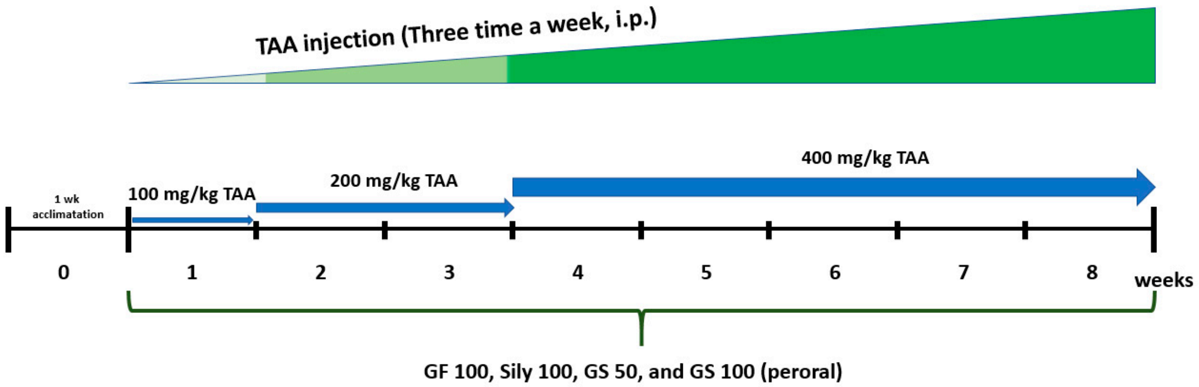

Supplementary Figure S1. Experimental schedule for animal experiment. During 8 weeks of the entire experiment period, we orally administrated with distilled water, Gardeniae Fructus (100 mg/kg, GF 100), Silymarin (100 mg/kg, Sily 100), GF and Silymarin mixtures (50 and 100 mg/kg, GS 50 and 100). For liver fibrosis, thioacetamide was intraperitoneally injected as 100 mg/kg for the first week, 200 mg/kg from second to third week, and 400 mg/kg from fourth to eighth week, respectively.

Supplementary Figure S2.

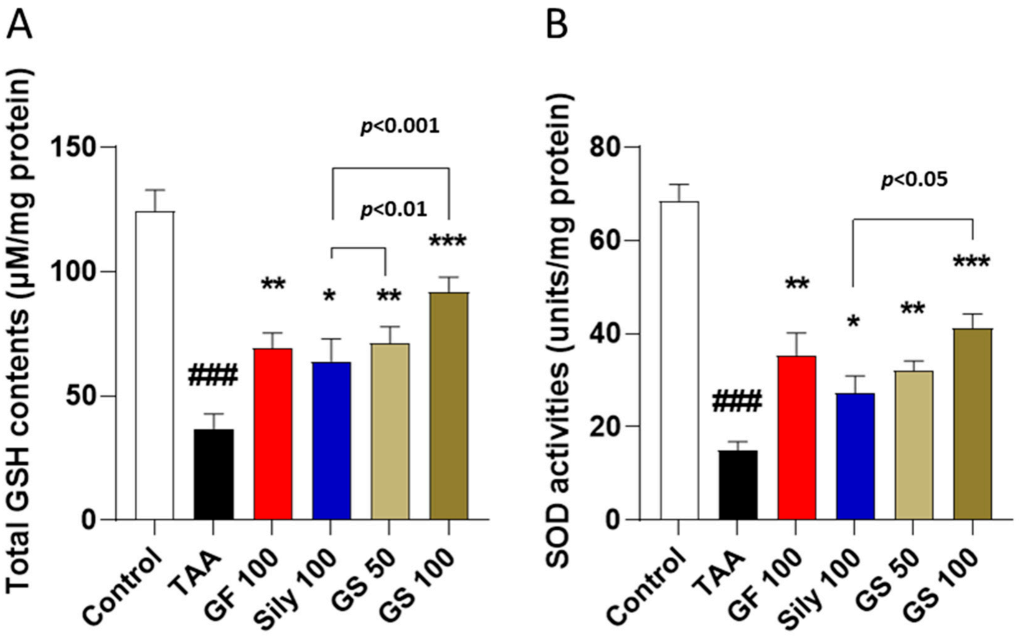

Supplementary Figure S2. Synergistic effects of GS on the antioxidant capacities against TAA-induced liver fibrosis. (A) Total GSH contents and (B) SOD activities. Data were expressed by mean ± S.E.M. $^{###}$p<0.001 for Control vs. TAA. *p<0.05, **p<0.01, and ***p<0.001 for TAA vs. drug treatments (n=6-8 for biochemistry analysis).

Supplementary Figure S3.

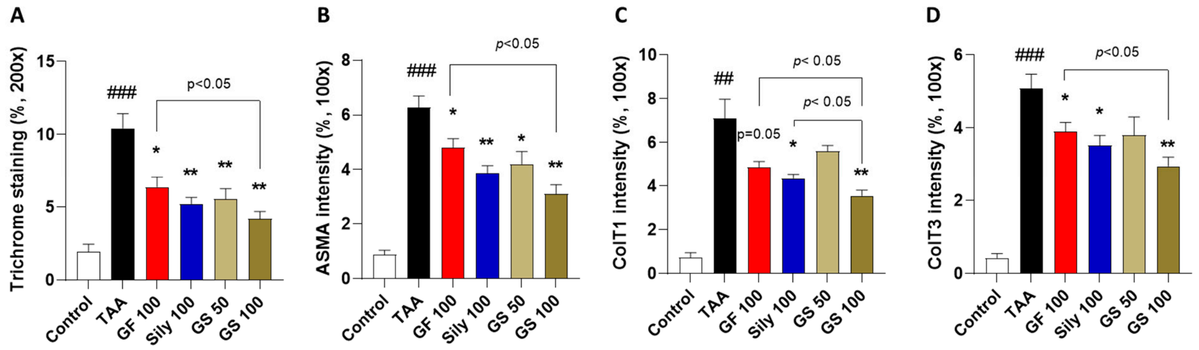

Supplementary Figure S3. Quantitative analysis of trichrome staining and IHC analysis. Quantitative analysis of each staining was happened by (A) trichrome staining, (B) IHC against ASMA, (C) ColT1, and (D) ColT3, respectively. Data were expressed by mean ± S.E.M. $^{\#\#}p<0.01$ and . $^{\#\#\#}p<0.001$ for Control vs. TAA. $^{*}p<0.05$ and $^{**}p<0.01$ for TAA vs. drug treatments. Images were captured by light microscopy condition (200x for trichrome staining and 100x for IHC analysis).

Supplementary Figure S4.

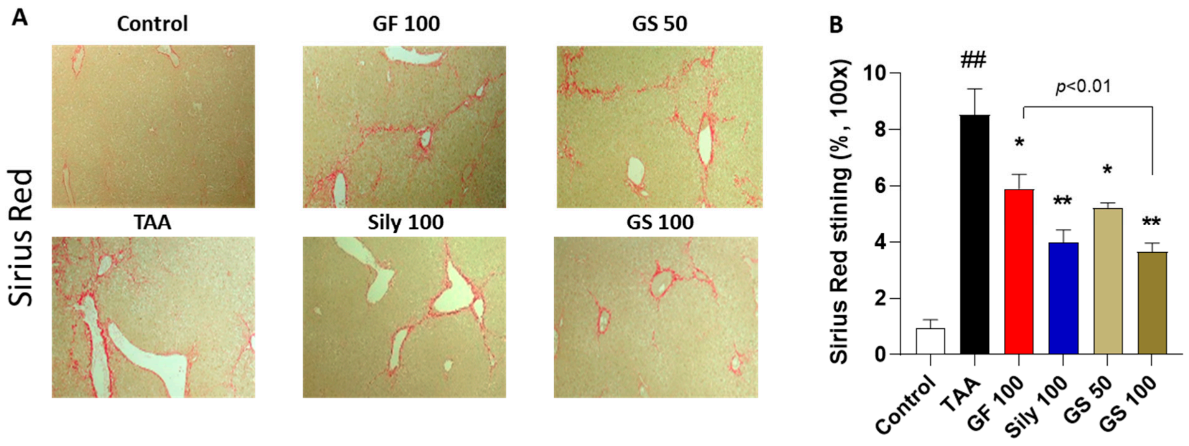

Supplementary Figure S4. Sirius Red staining and its quantitative analysis. (A) Representative images of Sirius Red staining in the liver tissue and (B) quantitative analysis of Sirius Red staining. Data were expressed by mean ± S.E.M. $^{##}p<0.01$ for Control vs. TAA. $^{*}p<0.05$ and $^{**}p<0.01$ for TAA vs. drug treatments. Images were captured by light microscopy condition (100x).

Supplementary Figure S5.

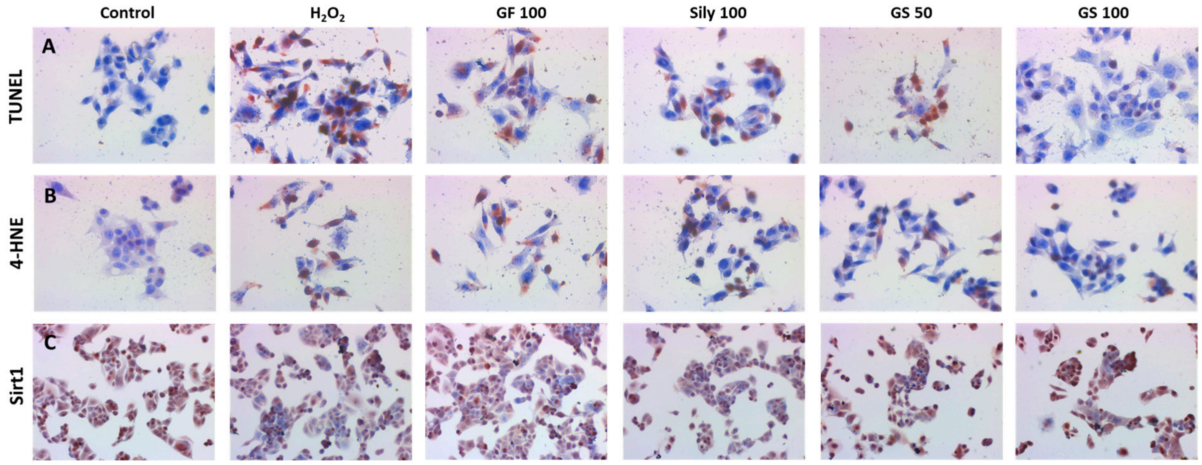

Supplementary Figure S5. Antioxidant effects of GS on the oxidative stress-mediated hepatocyte cell deaths. (A) Representative images of TUNEL assay. (B) IHC analysis against 4-HNE and (C) Sirt1 was performed. Images were captured by light microscopy condition (100x for Sirt1; 400x for TUNEL and 4-HNE).

Supplementary Figure S6.

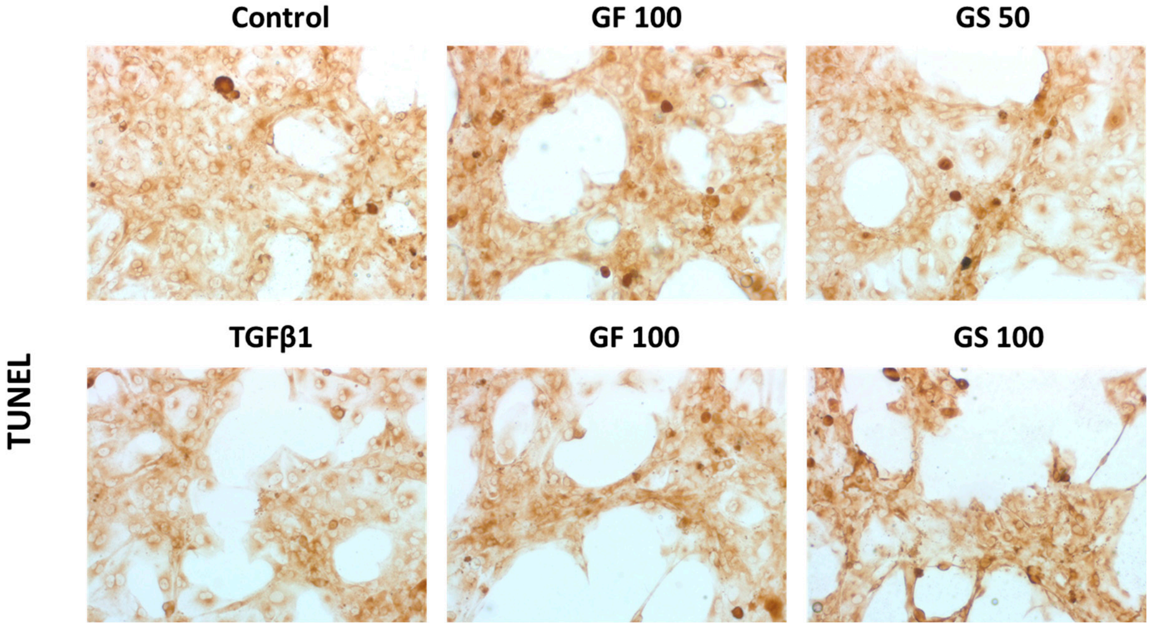

Supplementary Figure S6. GS promotes activated HSCs cell death during fibrotic stimulus. Representative images of TUNEL assay. Images were captured by light microscopy condition (400x for TUNEL).
