# Supplementary material for: Co-Treatments of Gardeniae Fructus and Silymarin Ameliorates Excessive Oxidative Stress-Driven Liver Fibrosis by Regulation of Hepatic Sirtuin1 Activities Using Thioacetamide-Induced Mice Model
Source: Antioxidants (Basel). 2022 Dec 30;12(1):97. doi: 10.3390/antiox12010097 (PMC9854785; doi:10.3390/antiox12010097)
Supplement: Supplementary file 1 [file antioxidants-12-00097-s001.zip › Supplementary Table S1. Antibody lists for Western blot analysis.pdf]

Supplementary Table S1. Antibody lists for Western blot analysis.

Antibody	Company	Cat. No	Dilution
GPx-4	Protein Tech	Cat #14432-1-AP	1:1000
SOD2	Santa Cruz Biotechnology	SC-17767	1:1000
4-HNE	R&D Systems	MAB3249	1:1000
p-NF-κB	Cell Signaling Technology	3033S	1:1000
NF-κB	Santa Cruz Biotechnology	SC-8008	1:1000
IκBα	Santa Cruz Biotechnology	SC-1643	1:1000
iNOS	Santa Cruz Biotechnology	SC-7271	1:1000
TLR4	Invitrogen	MA5-16216	1:1000
TGF-βR1	Novus	RM0016-3A11	1:1000
TIMP-1	Santa Cruz Biotechnology	SC-21734	1:1000
MMP-13	Abcam	ab39012	1:1000
SIRT1	Cell Signaling Technology	8469S	1:500
H3K9Ac	Cell Signaling Technology	7538S	1:500
H3K56Ac	Cell Signaling Technology	4243S	1:500
Total H3	Cell Signaling Technology	9717S	1:1000
p-AMPKα	Cell Signaling Technology	#2531	1:1000
AMPKα	Cell Signaling Technology	#2532	1:1000
HO-1	Santa Cruz Biotechnology	SC-136960	1:1000
Nrf2	Santa Cruz Biotechnology	SC-365949	1:1000
Collagen I	Abcam	ab34710	1:1000
Collagen III	Protein Tech	22734-1-AP	1:1000
α-SMA	Abcam	ab5694	1:1000
pSmad2	Cell Signaling Technology	18338S	1:1000
Smad2	Cell Signaling Technology	5339S	1:1000
pSmad3	Cell Signaling Technology	9520S	1:1000
Smad3	Cell Signaling Technology	9523S	1:1000

β-actin	Santa Cruz Biotechnology	SC-4778	1:1000
α-actinin	Santa Cruz Biotechnology	sc-390205	1:3000
HRP-conjugated anti-mouse IgG	GeneTex	GTX213110-01	1:3000
HRP-conjugated anti-rabbit IgG	GeneTex	GTX213111-01	1:3000
