# Supplementary material for: Co-Treatments of Gardeniae Fructus and Silymarin Ameliorates Excessive Oxidative Stress-Driven Liver Fibrosis by Regulation of Hepatic Sirtuin1 Activities Using Thioacetamide-Induced Mice Model
Source: Antioxidants (Basel). 2022 Dec 30;12(1):97. doi: 10.3390/antiox12010097 (PMC9854785; doi:10.3390/antiox12010097)
Supplement: Supplementary file 1 [file antioxidants-12-00097-s001.zip › Supplementary Table S2. Antibody lists for immunohistochemistry and immunofluorescence analysis.pdf]

Supplementary Table S2. Antibody lists for immunohistochemistry and immunofluorescence analysis.

| **Antibody** | **Company** | **Cat. No** | **Dilution** |
|---|---|---|---|
| 4-HNE | Santa Cruz Biotechnology | SC-133160 | 1:1000 |
| F4/80 | Abcam | SC-17767 | 1:1000 |
| 8-OHdG | Bioss | BS-1278R | 1:100 |
| Collagen 1 | Abcam | ab34710 | 1:200 |
| SIRT 1 | Cell signaling technology | 8469S | 1:100 |
| SIRT 1 | Cell signaling technology | 8469S | 1:100 |
